# Longitudinal Synaptic Loss in Primary Tauopathies: An In Vivo [ ^11^C]UCB‐J Positron Emission Tomography Study

**DOI:** 10.1002/mds.29421

**Published:** 2023-05-12

**Authors:** Negin Holland, P. Simon Jones, George Savulich, Michelle Naessens, Maura Malpetti, David J. Whiteside, Duncan Street, Peter Swann, Young T. Hong, Tim D. Fryer, Timothy Rittman, Eoin Mulroy, Franklin I. Aigbirhio, Kailash P. Bhatia, John T. O'Brien, James B. Rowe

**Affiliations:** ^1^ Department of Clinical Neurosciences University of Cambridge, Cambridge Biomedical Campus Cambridge United Kingdom; ^2^ Cambridge University Hospitals NHS Foundation Trust Cambridge United Kingdom; ^3^ Department of Psychiatry University of Cambridge, School of Clinical Medicine, Cambridge Biomedical Campus Cambridge United Kingdom; ^4^ Wolfson Brain Imaging Centre University of Cambridge Cambridge United Kingdom; ^5^ Department of Clinical and Movement Neurosciences UCL Queen Square Institute of Neurology London United Kingdom; ^6^ Medical Research Council Cognition and Brain Sciences Unit University of Cambridge Cambridge United Kingdom

**Keywords:** [^11^C]UCB‐J PET, primary tauopathies, longitudinal

## Abstract

**Background:**

Synaptic loss is characteristic of many neurodegenerative diseases; it occurs early and is strongly related to functional deficits.

**Objective:**

In this longitudinal observational study, we determine the rate at which synaptic density is reduced in the primary tauopathies of progressive supranuclear palsy (PSP) and corticobasal degeneration (CBD), and we test the relationship with disease progression.

**Methods:**

Our cross‐sectional cohort included 32 participants with probable PSP and 16 with probable CBD (all amyloid‐negative corticobasal syndrome), recruited from tertiary care centers in the United Kingdom, and 33 sex‐ and age‐matched healthy control subjects. Synaptic density was estimated by positron emission tomography imaging with the radioligand [^11^C]UCB‐J that binds synaptic vesicle 2A. Clinical severity and cognition were assessed by the PSP Rating Scale and the Addenbrooke's cognitive examination. Regional [^11^C]UCB‐J nondisplaceable binding potential was estimated in Hammersmith Atlas regions of interest. Twenty‐two participants with PSP/CBD had a follow‐up [^11^C]UCB‐J positron emission tomography scan after 1 year. We calculated the annualized change in [^11^C]UCB‐J nondisplaceable binding potential and correlated this with the change in clinical severity.

**Results:**

We found significant annual synaptic loss within the frontal lobe (−3.5%, *P* = 0.03) and the right caudate (−3.9%, *P* = 0.046). The degree of longitudinal synaptic loss within the frontal lobe correlated with the rate of change in the PSP Rating Scale (*R* = 0.47, *P* = 0.03) and cognition (Addenbrooke's Cognitive Examination–Revised, *R* = −0.62, *P* = 0.003).

**Conclusions:**

We provide in vivo evidence for rapid progressive synaptic loss, correlating with clinical progression in primary tauopathies. Synaptic loss may be an important therapeutic target and outcome variable for early‐phase clinical trials of disease‐modifying treatments. © 2023 The Authors. *Movement Disorders* published by Wiley Periodicals LLC on behalf of International Parkinson and Movement Disorder Society.

## 1 Introduction

The primary tauopathies of progressive supranuclear palsy (PSP) and corticobasal degeneration (CBD) are devastating and rapidly progressive neurodegenerative diseases with a poor prognosis and common diagnostic delays.^1^, ^2^ The pathologies of PSP and CBD cause diverse clinical syndromes, with a combination of movement and cognitive impairment,^3^, ^4^, ^5^, ^6^ including clinical PSP and the corticobasal syndrome (CBS; in the absence of Alzheimer's disease pathology, CBD pathology is the most common underlying cause of the CBS).^7^ Pathologically, PSP and CBD are associated with accumulation of hyperphosphorylated 4‐repeat tau,^8^, ^9^, ^10^, ^11^ inflammation,^12^, ^13^, ^14^ and severe synaptic loss,^15^, ^16^, ^17^ ultimately all leading to neuronal loss. Cognitive dysfunction is an early symptom in people with PSP/CBD, with subtle manifestations apparent 8 years before diagnosis.^18^

Synaptic dysfunction is proposed as a key pathogenic mechanism underlying cognitive dysfunction,^19^, ^20^ via changes in network physiology.^21^ In preclinical tauopathy models, severe synaptic loss can occur before neuronal loss.^22^ As such, the pathways involved in synaptic function, plasticity, and stability are of importance for understanding the early phases of neurodegeneration and as potential early therapeutic targets. It is encouraging that in the alternative tauopathy of Alzheimer's disease, in vivo synaptic imaging, with [^11^C]UCB‐J positron emission tomography (PET), is sensitive to progressive changes in synaptic density and response to early‐phase trials, for example, the synaptic modulator saracatinib^23^ and the modulator of synaptic vesicle glycoprotein 2A SDI‐118.^24^

The aim of this study was to test the hypothesis that cognitive and functional decline in PSP/CBD is related to progressive synaptic loss. Cryoelectron microscopy studies and astrocytic pathology indicate differences between PSP and CBD pathology.^11^ However, we consider them jointly here because of downstream convergence in secondary pathological processes and overlapping clinical features.^25^ We used [^11^C]UCB‐J PET, which targets presynaptic vesicle glycoprotein SV2A, to estimate synaptic density in vivo.^26^ Using this PET radioligand, we recently reported a widespread baseline in vivo synaptic loss (up to 30%) in a smaller cross‐sectional cohort of patients with PSP and CBD. The most severe reductions were in the basal ganglia, thalamus, insula, and frontal and temporal lobes, and these correlated with cognition and disease severity.^17^ Based on the average symptom duration of 4 to 6 years in that study, we predicted that synaptic loss in PSP/CBD progresses at a rate of ~3% to 4% per year, with faster synaptic loss being seen in basal ganglia and frontal cortex.^9^

## 2 Subjects and Methods

### 2.1 Participants and Study Design

#### 2.1.1 Recruitment

For the cross‐sectional cohort, we used the 2017 diagnostic criteria for patients with probable PSP–Richardson syndrome^5^ and the Armstrong criteria for patients with probable CBS.^4^ Thirty‐two people with probable PSP–Richardson syndrome and 25 people with probable CBS were recruited from a regional specialist National Health Service clinic at the Cambridge University Center for Parkinson‐plus; people with CBS were also recruited from the National Hospital for Neurology and Neurosurgery at Queen Square, London. Patients with CBS underwent amyloid PET imaging using Pittsburgh Compound B ([^11^C]PiB). Only those with a negative amyloid status (16/25 participants) are included in subsequent analysis, because we aimed to exclude patients with CBS caused by Alzheimer's disease. We interpret and refer to the amyloid‐negative CBS cohort as having CBD, although we acknowledge that other pathologies are possible.^7^ Thirty‐three healthy volunteers were recruited from the UK National Institute for Health Research Join Dementia Research register. Participants were screened using the inclusion/exclusion criteria set out in Holland et al.^17^ Fourteen of 32 patients had passed away by study endpoint (August 2022), with nine brains subsequently donated to our local Cambridge Brain Bank. Three of nine are confirmed as having PSP at postmortem, with more recent donations awaiting neuropathological confirmation.

For the follow‐up cohort, 22 participants (16 with PSP and 6 with CBD) remained well enough over 1 year to be reassessed in the longitudinal arm of the study.

#### 2.1.2 Neurocognitive and Imaging Assessments

Eligible participants underwent clinical and cognitive assessments (Table 1), as previously described by Holland et al.^17^ Participants underwent simultaneous 3‐T magnetic resonance imaging (MRI) and [^11^C]UCB‐J PET. Our follow‐up cohort underwent repeat MRI, [^11^C]UCB‐J PET, and neurocognitive assessment at 1 year.

**TABLE 1:** Demographics and clinical characteristics

Parameters	Control‐N	PSP‐N	CBD‐N	F(*P*)^a^	PSP‐BL	CBD‐BL	F(*P*)^b^	PSP‐FU	CBD‐FU	F(*P*)^c^
N	31	16	10	–	16	6	–	16	6	–
Male:female	20:11	8:8	6:4	–	7:9	2:4	–	7:9	2:4	–
Age at baseline (y)	71.0 (8.5)	71.7 (8.24	70.7 (7.2)	ns	72.1 (8.1)	71 (10.8)	ns	–	–	–
Education (y)	14.4 (3.4)	11.4 (4.0)	12.5 (2.5)	**5**.**7** (***0***. ***005***)	11.1 (2.3)	12 (2.5)	ns	–	–	–
Symptom duration (y)	–	4.15 (2.3)	4.3 (2.8)	ns	4.2 (1.9)	5.2 (4.1)	ns	5.4 (1.8)	6.4 (4.1)	–
UCB‐J scan interval at FU (months)	–	–	–	–	–	–	–	13.8 (5.5)	14 (4.3)	–
UCB‐J injected activity (MBq)	306 (129)	268 (107)	271 (89)	ns	334 (105)	293 (66)	ns	262 (84)	258 (74)	ns
Total MMSE (max. 30)	29.5 (1.1)	27.0 (3.4)	25.0 (4.6)	**9**.**5** (**<*0***. ***001***)	27.6 (2.2)	24.5 (5.8)	ns	26.4 (5.6)	24.6 (3.4)	ns
Total ACE‐R (max. 100)	96.3 (2.7)	80.8 (13.4)	76.8 (15.4)	**20**.**3** (**<0**.**0001**)	84.3 (8.3)	69.7 (19.7)	ns	73.9 (18.7)	74.8 (8.8)	ns
Attention + Orientation (max. 18)	17.9 (0.3)	16.3 (2.8)	16.1 (2.2)	**5**.**7** (**<*0***. ***01***)	16.9 (1.6)	15.5 (3)	ns	15.8 (2.2)	15.2 (2.3)	ns
Language (max. 26)	25.7 (0.8)	6.5 (3.4)	6.9 (3.45)	**6**.**2** (**<*0***. ***01***)	23.6 (2.1)	18.2 (8.8)	ns	21.1 (6.4)	23.4 (3.2)	ns
Fluency (max. 14)	12.4 (1.5)	23.2 (4.3)	21.6 (6.0)	**36**.**9** (**<*0***. ***0001***)	7.9 (2.5)	5.8 (3.3)	ns	5.9 (3.5)	6 (2.2)	ns
Memory (max. 26)	24.6 (1.9)	21.7 (3.7)	20.5 (4.7)	**8**.**6** (**<*0***. ***01***)	22.1 (3.4)	19.2 (3.8)	ns	19.7 (5.2)	18.4 (3.8)	ns
Visuospatial (max. 16)	15.6 (0.6)	13.1 (3.1)	11.8 (4.5)	**11**.**1** (**<*0***. ***0001***)	13.8 (2.1)	11 (6.3)	ns	10.9 (4.3)	11.8 (2.8)	ns
Total PSP Rating Scale	‐	34.0 (9.7)	26.4 (13.0)	ns	29.5 (8.5)	34.8 (12.7)	ns	44.6 (16.8)	50.2 (19.3)	**15**.**5** (**<*0***. ***01***)
Total CBI	‐	47.4 (34.0)	37.8 (21.4)	ns	44 (33.2)	39 (8)	ns	58.7 (33.4)	46.7 (19.8)	ns
Total CDR	‐	8.5 (5.1)	6.9 (5.2)	ns	5.6 (3.8)	7 (4.3)	ns	10.6 (4.1)	8.5 (5.3)	**10**.**3 (<0**.**01)**

*Note*: The results are given as mean (standard deviation). The F‐statistic and *P* values are derived from ANOVA. Significant values are expressed in bold italics.

^a^ ANOVA between patients and control subjects.

^b^ ANOVA between N and BL patient cohorts.

^c^ Paired ANOVA between BL and FU assessment. *P‐values* < 0.05 after multiple comparison correction (false discovery rate).

Abbreviations: N, neurocognitive profile of participants who competed only the cross‐sectional study; PSP, progressive supranuclear palsy–Richardson's syndrome; CBD, corticobasal syndrome with a negative amyloid biomarker from [^11^C]PiB PET; BL, baseline neurocognitive profile of participants who came to follow‐up; FU, neurocognitive profile of patients at follow‐up; ns, not significant; MMSE, Mini‐Mental State Examination; max., maximum; ACE‐R, Addenbrooke's Cognitive Examination–Revised; CBI, revised Cambridge Behavioral Inventory; CDR, Clinical Dementia Rating Scale; PET, positron emission tomography; ANOVA, analysis of variance.

The research protocol was approved by the Cambridge Research Ethics Committee (reference 18/EE/0059) and the UK Administration of Radioactive Substances Advisory Committee. All participants provided written informed consent in accordance with the Declaration of Helsinki.

### 2.2 Imaging Data Acquisition and Preprocessing

#### 2.2.1 PET and MRI

We first corroborated our cross‐sectional findings in Holland et al^17^ with this larger cohort of healthy control and patient participants. The procedures for [^11^C]UCB‐J synthesis, PET data acquisition, image reconstruction, and kinetic analysis were the same as in Holland et al.^17^ In brief, dynamic PET data acquisition was performed on a GE SIGNA PET/MR (GE Healthcare, Waukesha, WI, USA) for 90 minutes immediately after injection, with attenuation correction using a multisubject atlas method^27^ and improvements to the MRI brain coil component.^28^ Emission image series were aligned using SPM12 (www.fil.ion.ucl.ac.uk/spm/software/spm12/) and rigidly registered to the contemporaneously acquired T1‐weighted MRI during PET data acquisition, allowing for more accurate coregistration and reduced scanning sessions from two separate scans to one combined session (repetition time = 3.6 ms, echo time = 9.2 ms, 192 sagittal slices, in‐plane resolution 0.55 × 0.55 mm, interpolated to 1.0 × 1.0 mm; slice thickness 1.0 mm). The Hammersmith atlas (http://brain-development.org) with modified posterior fossa regions was nonrigidly registered to the T1‐weighted MRI of each participant using spatial normalization parameters determined with Advanced Normalization Tools (ANTs) software.^29^

##### 2.2.1.1 Regional Analysis

Regional time‐activity curves were extracted after the application of cerebrospinal fluid (CSF) partial volume correction (PVC) to each dynamic PET image. To assess the impact of PVC, we also extracted time‐activity curves from the same region of interests without the application of PVC (discussed in Supporting Information as “without partial volume correction”).

To quantify SV2A density, we determined [^11^C]UCB‐J nondisplaceable binding potential (BP_ND_) using a basis function implementation of the simplified reference tissue model,^30^ with the reference tissue defined in the centrum semiovale.^31^, ^32^ [^11^C]UCB‐J BP_ND_ from the following Hammersmith atlas regions was not used: lateral ventricles (frontal and temporal horns), third ventricle, pituitary gland, and the corpus callosum. We excluded two subcortical gray matter regions from the PET analysis (substantia nigra, nucleus accumbens) because of their very small volume. Three participants from the follow‐up cohort (two with PSP and one with CBD) had a reduced emission duration of 60 minutes at follow‐up. For these individuals, their baseline binding potentials were recalculated using 60 minutes (rather than 90 minutes) to eliminate any duration‐induced bias in the comparison of baseline and follow‐up BP_ND_ values.

Gray matter volumes were extracted from the unsmoothed gray matter segmentation maps from SPM12 within each Hammersmith regions of interest in native space, at baseline and follow‐up. All regions, except those in the earlier‐mentioned “excluded” regions, were taken forward to the subsequent statistical analyses.

##### 2.2.1.2 Voxelwise analysis

The T1‐weighted images were first processed using the Computational Anatomy Toolbox (CAT12.8.1; http://www.neuro.uni-jena.de/cat12) and the Segment Longitudinal Data module of SPM12 (v.7771; www.fil.ion.ucl.ac.uk/spm/software/spm12/). A pairwise unbiased pipeline was performed using the T1‐weighted images to bring all images into a standard space, including [^11^C]UCB‐J BP_ND_ maps produced using the same kinetic modeling approach as for the regional analysis. The T1‐weighted image pairs were iteratively warped to produce single‐subject templates, which were subsequently processed with CAT12 Segment. Next, the tissue segments were applied to the templates to create skull‐stripped single‐subject templates, which were warped to a group average space. The group template was warped to the MNI152NL in 2009 Asym template in CAT12 (MNI, Montreal Neurological Institute). Finally, all warps and linear transformations were combined, and Jacobians were created to enable the gray matter images to be modulated and warped to MNI space. The [^11^C]UCB‐J BP_ND_ images were linearly coregistered to the contemporary T1‐weighted images using FreeSurfer's mri_coreg function. Each output linear transformation matrix was converted to ITK (Interoperability Toolkit) format, which could be read by ANTs tools. This transform was combined with the warps and linear transforms created in the ANTs pipeline to place the [^11^C]UCB‐J BP_ND_ images in MNI space.

##### 2.2.1.3 [^11^C]PiB PET

Amyloid imaging using [^11^C]PiB followed the protocol given in Holland et al.^17^ A negative amyloid status was characterized by a cortical [^11^C]PiB standardized uptake value ratio of <1.21.^33^

### 2.3 Statistical Analyses

#### 2.3.1 Regional

We first corroborated the results in Holland et al^17^ using a larger cross‐sectional cohort. We compared demographic and clinical variables between patients and control subjects in this larger sample, using analysis of covariance and chi‐square tests where appropriate. We also presented the baseline characteristics of the 22 participants who completed the longitudinal arm of the study separately and compared their demographics with patients who completed only the cross‐sectional arm. We compared cross‐sectional regional [^11^C]UCB‐J BP_ND_ between patients and control subjects using analysis of covariance with age and total intracranial volume in the case of gray matter volume comparisons as covariates of no interest, adjusting for multiple comparisons (false discovery rate method; results for analyses without PVC are shown in the Supporting Information in Data S1).

In the longitudinal cohort, the proportional change in [^11^C]UCB‐J BP_ND_ was calculated as follows: proportional change in [^11^C]UCB‐J BP_ND_ = (follow‐up UCB‐J BP_ND_ – baseline UCB‐J BP_ND_)/baseline UCB‐J BP_ND_. Proportional changes in [^11^C]UCB‐J BP_ND_ were adjusted for the time interval between [^11^C]UCB‐J PET scans. Although most patients were rescanned ~12 months after baseline, some were scanned later because of the COVID‐19 pandemic—proportional changes are therefore presented as the annualized proportional change in [^11^C]UCB‐J BP_ND_.

A one‐sample *t* test was used across all the regional annualized proportional changes in [^11^C]UCB‐J BP_ND_, testing whether the overall mean change was smaller than zero (ie, synapse loss). Next, a two‐way paired‐sample analysis of variance (ANOVA) design was used to test for any region‐by‐visit interaction with visit (baseline vs. follow‐up) and region as within‐subject variables; a post hoc analysis was carried out to interrogate any significant interactions (correcting for multiple comparisons using the false discovery rate).

Given the smoothness and expected spatial contiguity of pathology, we performed a principal‐component analysis to identify principal components that correlated with changes in neurocognitive measures over time (including the PSP Rating Scale and the Addenbrooke's Cognitive Examination–Revised [ACE‐R]). The same approach was used to assess changes in gray matter volume over time. Principal‐component analysis captures the majority of variance in a low‐dimensional space, minimizing the problem of multiple comparisons and providing greater clarity in spatial patterns of pathology/atrophy.

Statistical analyses were implemented in R (version 4.2.0; R Core Team 2021; https://www.R-project.org/).

#### 2.3.2 Voxelwise Analysis Statistics

The warped, modulated gray matter image differences were smoothed (8‐mm full‐width at half‐maximum), divided by the interscan interval, and subsequently compared using a one‐sample *t* test in FSL randomized (FSL v6.0.5.1; fsl.fmrib.ox.ac.uk) with threshold‐free cluster enhancement and 10,000 permutations. Clusters were deemed significant at the *P* < 0.05 corrected level. In addition, covariate analyses (PSP Rating Scale and the ACE‐R) were performed comparing the difference in covariate score with the gray matter difference images. The analysis was repeated for the [^11^C]UCB‐J BP_ND_ images by creating BP_ND_ difference images between baseline and follow‐up. Images were scaled by the group mean global BP_ND_ across both time points to reduce the level of noise. To match the smoothness of the gray matter on MRI, we smoothed BP_ND_ images by a further 5.3‐mm full‐width at half‐maximum.

## 3 Results

### 3.1 Demographics

We observed typical cognitive profiles in the cross‐sectional cohort of patients (who completed only the cross‐sectional arm of the study): patients were impaired on memory, verbal fluency, language and visuospatial domains of the ACE‐R, and the Mini‐Mental State Examination. There were high endorsements on the Cambridge Behavioral Inventory, and high scores on the Clinical Dementia Rating scale sum of boxes. The baseline neurocognitive profile of the 22 patients in the longitudinal arm (16 with PSP and 6 with CBD) was similar to those patients who completed only the cross‐sectional arm of the study. Importantly, the two groups did not differ in their average symptom duration or severity (Table 1). Within our longitudinal cohort, the average time interval between the baseline and follow‐up scan was 13.8 (±5.5) and 12.0 (±4.3) months in the PSP and CBD cohorts, respectively. Our longitudinal cohort scored higher on both the PSP and the Clinical Dementia Rating Scales at follow‐up: mean increases of 13.6 (±10.2) and 4.33 (±3.15), respectively. The average annual decline in the ACE‐R score was −6.9 points (±14.5).

### 3.2 Widespread Severe Reductions in Regional [ ^11^C]UCB‐J BP_ND_ Beyond Gray Matter Atrophy: Cross‐Sectional Cohort

**FIG 1:**
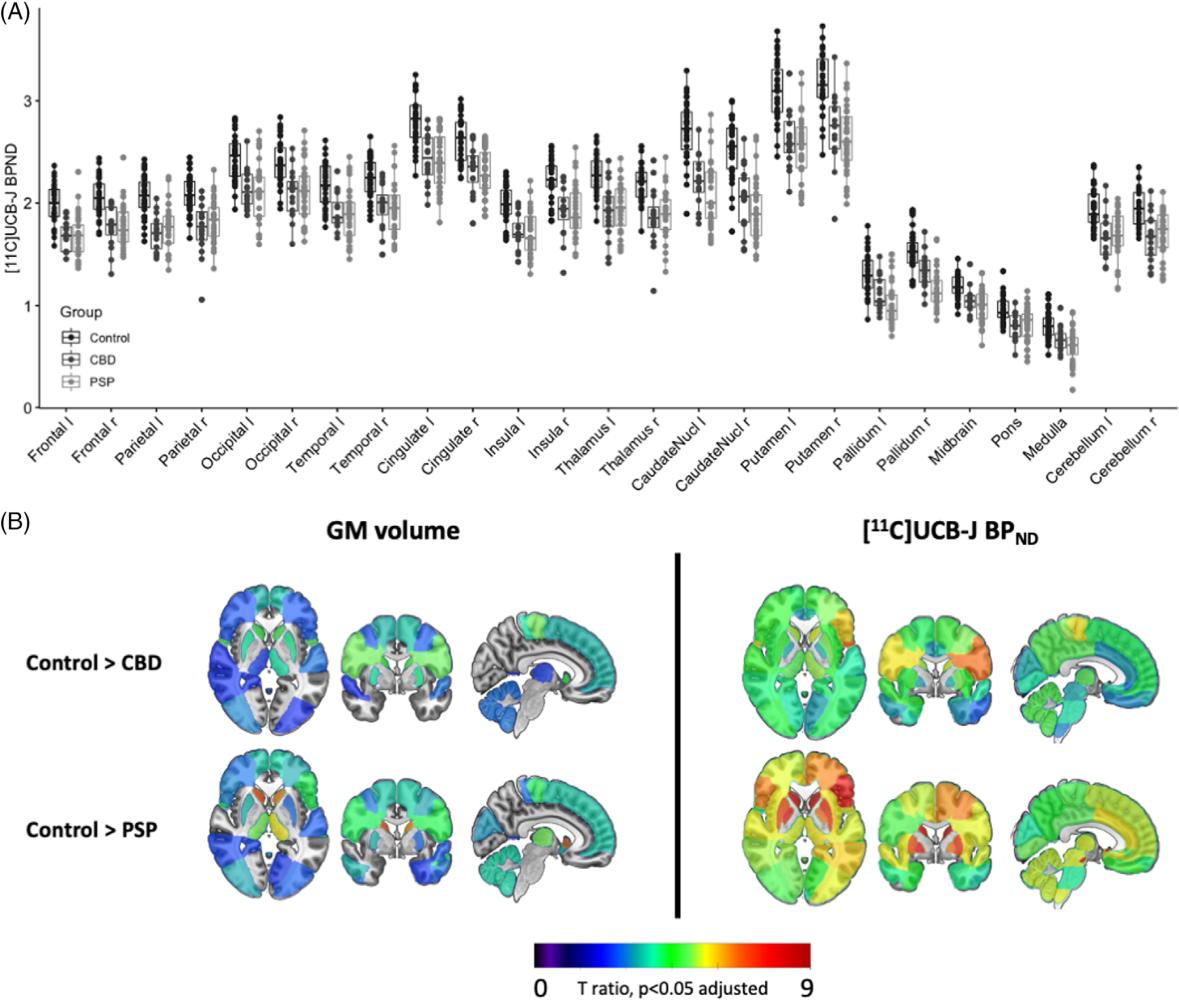
(**A**) Baseline regional [^11^C]UCB‐J BP_ND_ in healthy volunteers (n = 31) and patients (progressive supranuclear palsy [PSP], n = 32; corticobasal degeneration [CBD], n = 16) in major regions of interest. (**B**) *t*‐Statistic maps comparing regional gray matter (GM) volume and [^11^C]UCB‐J BP_ND_ (CSF corrected) in patients versus controls. Only *t* values, significant at *P* < 0.05 adjusted for multiple comparisons, are shown; higher *t* values illustrate greater volume and synaptic loss; orange/red = more severe atrophy/loss). [Color figure can be viewed at wileyonlinelibrary.com]

Synaptic loss was widespread across all major cortical and subcortical areas; [^11^C]UCB‐J binding potentials are shown in Figure 1A, and *t* statistics comparing synaptic density and gray matter volume against controls at adjusted *P* < 0.05 are shown in Figure 1B. Mean regional *Z* scores were calculated against the control cohort. In CBD, severe reductions were seen in the thalamus (mean *Z* score −1.8), parietal lobe (mean *Z* score −1.8), caudate (mean *Z* score −1.6), frontal lobe (mean *Z* score −1.6), cerebellum (mean *Z* score −1.6), and putamen (mean *Z* score −1.5). In PSP, severe reductions were seen in the caudate nucleus (mean *Z* score −2.1), pallidum (mean *Z* score −1.9), thalamus (mean *Z* score −1.6), frontal lobe (mean *Z* score −1.6), midbrain (mean *Z* score −1.6), and cingulate (mean *Z* score −1.6). Gray matter volume loss was also widespread but less severe (Fig. 1B). Mean *Z* scores for the most atrophied brain regions in CBD are putamen (−1.4), frontal lobe (−1.3), caudate nucleus (−1.3), and parietal lobe (−1.3); and in PSP are caudate nucleus (−1.5), thalamus (−1.3), frontal lobe (−1.1), and cerebellum (−1). Similar results were obtained using BP_ND_ without PVC (Supporting Information Fig. S1 in Data S1).

### 3.3 Longitudinal Changes in the PSP Rating Scale and the ACE‐R

**FIG 2:**
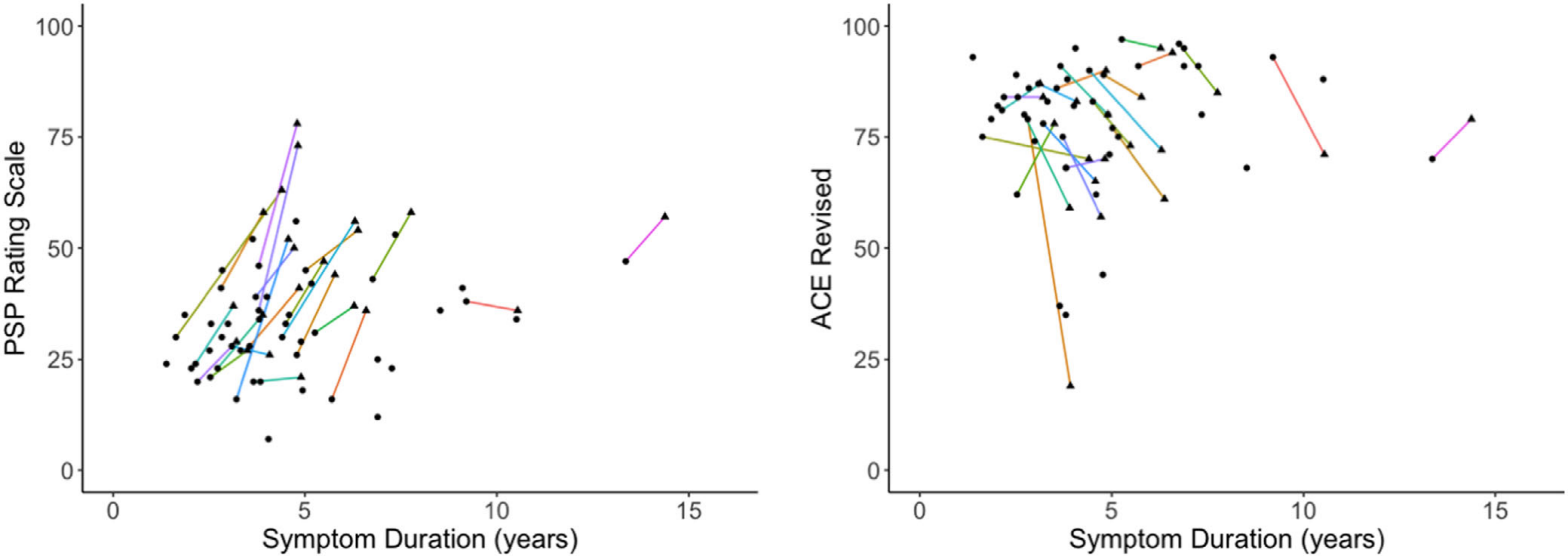
Progressive supranuclear palsy (PSP) Rating Scale on the left and Addenbrooke's Cognitive Examination–Revised (ACE‐R) scores on the right in 48 patients (PSP = 32, corticobasal degeneration [CBD] = 16) versus symptom duration, at baseline (circles) and at follow‐up (triangles) in 22 patients (PSP = 16, CBD = 6). Each colored line represents a patient who completed the follow‐up arm of the study. [Color figure can be viewed at wileyonlinelibrary.com]

Our patients presented with an average symptom duration of 4.6 years at baseline (range: 1.4–13.4 years), with an average score of 31.5 on the PSP Rating Scale (range: 7–56) and an average cognitive performance of 79.4 on the ACE‐R (range: 35–97) (circles in Fig. 2). Most patients had deteriorated cognitively and had more severe symptoms at follow‐up (triangles in Fig. 2). Our follow‐up patients had an average increase of 13.6 points on the PSP Rating Scale (range: −2.2 to 37) and a decline in their ACE‐R scores of −6.9 (range: −55.4 to 17.5) points over 1 year (Fig. 2).

### 3.4 Longitudinal Changes in [ ^11^C]UCB‐J BP_ND_ and Gray Matter Volume, and Correlations With Change in Symptom Severity Over Time

There was a significant overall reduction in [^11^C]UCB‐J BP_ND_ over 1 year (*P* = 0.013). A paired‐sample, two‐way ANOVA, testing for a difference in [^11^C]UCB‐J BP_ND_ between baseline and follow‐up visits, confirmed a significant region‐by‐visit interaction (*P* = 0.01). Post hoc paired‐sample ANOVA by region between visits confirmed significant reductions in [^11^C]UCB‐J BP_ND_ within the left presubgenual frontal cortex (−3.5%, *P* = 0.03) and the right caudate (−3.9%, *P* = 0.046), unadjusted; there was also a trend reduction in the left pallidum (−4.2%, *P* = 0.08). A summary of annual percentage change in [^11^C]UCB‐J BP_ND_ in all relevant Hammersmith atlas regions of interest is given in Supporting Information Table S1 in Data S1. The earlier analysis with gray matter volume also showed reductions over time; the most significant reductions were within the right thalamus (−11.8%, *P* = 0.002), precentral gyrus (−7.1%, *P* = 0.004), caudate nucleus (−7.5%, *P* = 0.009), and cerebellar gray matter (−2.8%, *P* = 0.04). A summary of regional gray matter annual percentage change is given in Supporting Information Table S2 in Data S1.

**FIG 3:**
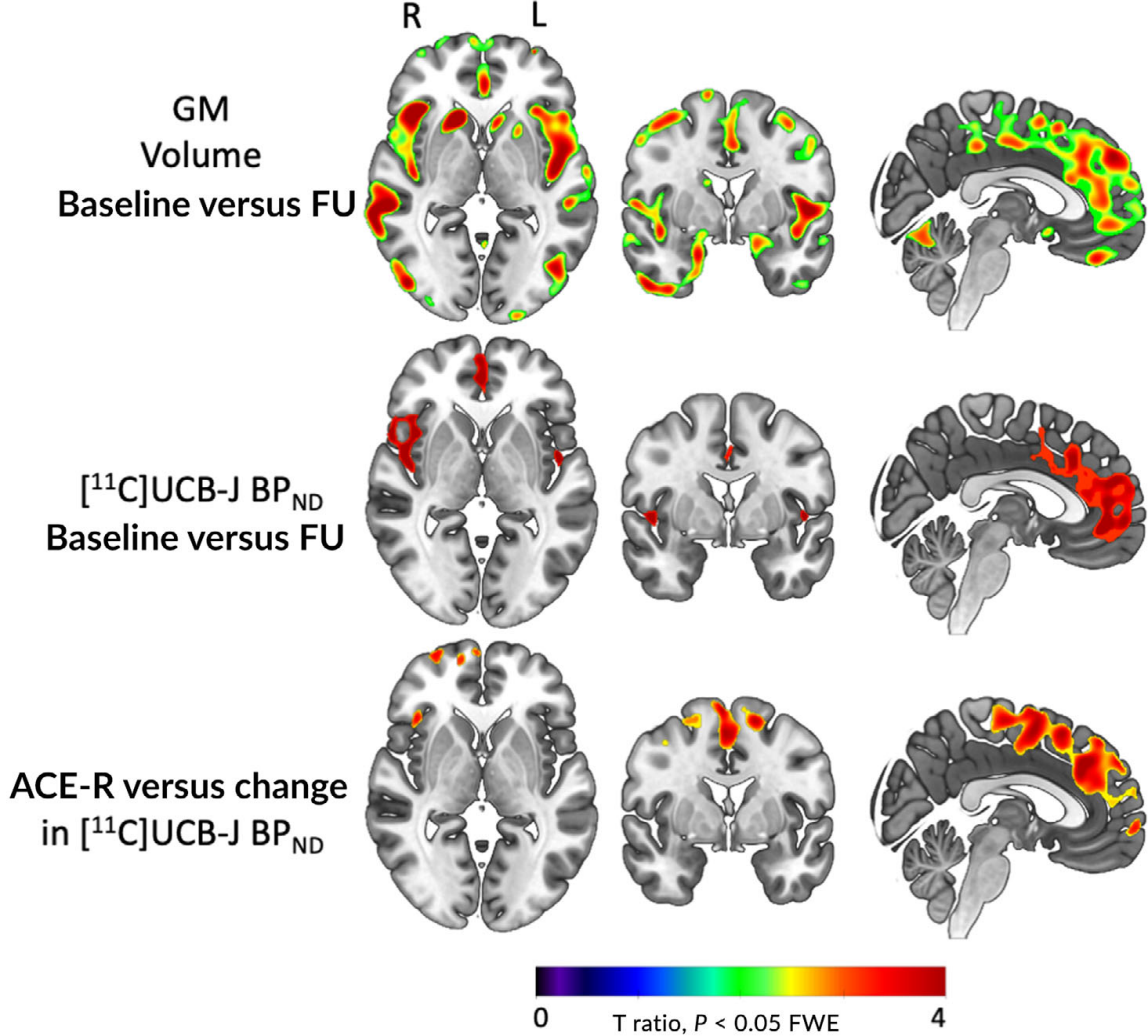
Voxelwise *t*‐ratio brain maps illustrating longitudinal change in gray matter volume and [^11^C]UCB‐J BP_ND_, and a significant relationship between reduced [^11^C]UCB‐J BP_ND_ and worse performance on the Addenbrooke's Cognitive Examination–Revised (ACE‐R) (*P* < 0.05 FWE corrected). FU, follow‐up; GM, gray matter. [Color figure can be viewed at wileyonlinelibrary.com]

Voxelwise results were in accord with the regional analysis. We confirm significant loss of gray matter volume and [^11^C]UCB‐J BP_ND_ at follow‐up, with the latter showing a more focal distribution but higher effect size than gray matter loss; *P* < 0.05, Family‐Wise Error (FWE) corrected. In voxelwise analysis, the correlation between gray matter loss and the PSP Rating Scale and ACE‐R scores was not significant. However, reductions in [^11^C]UCB‐J BP_ND_ within the frontal lobe were associated with poorer performance on the ACE‐R over time, but not the PSP Rating Scale (Fig. 3).

### 3.5 Faster Reductions in Frontal [ ^11^C]UCB‐J BP_ND_ Are Associated With Faster Symptom Progression: Principal‐Component Analysis

**FIG 4:**
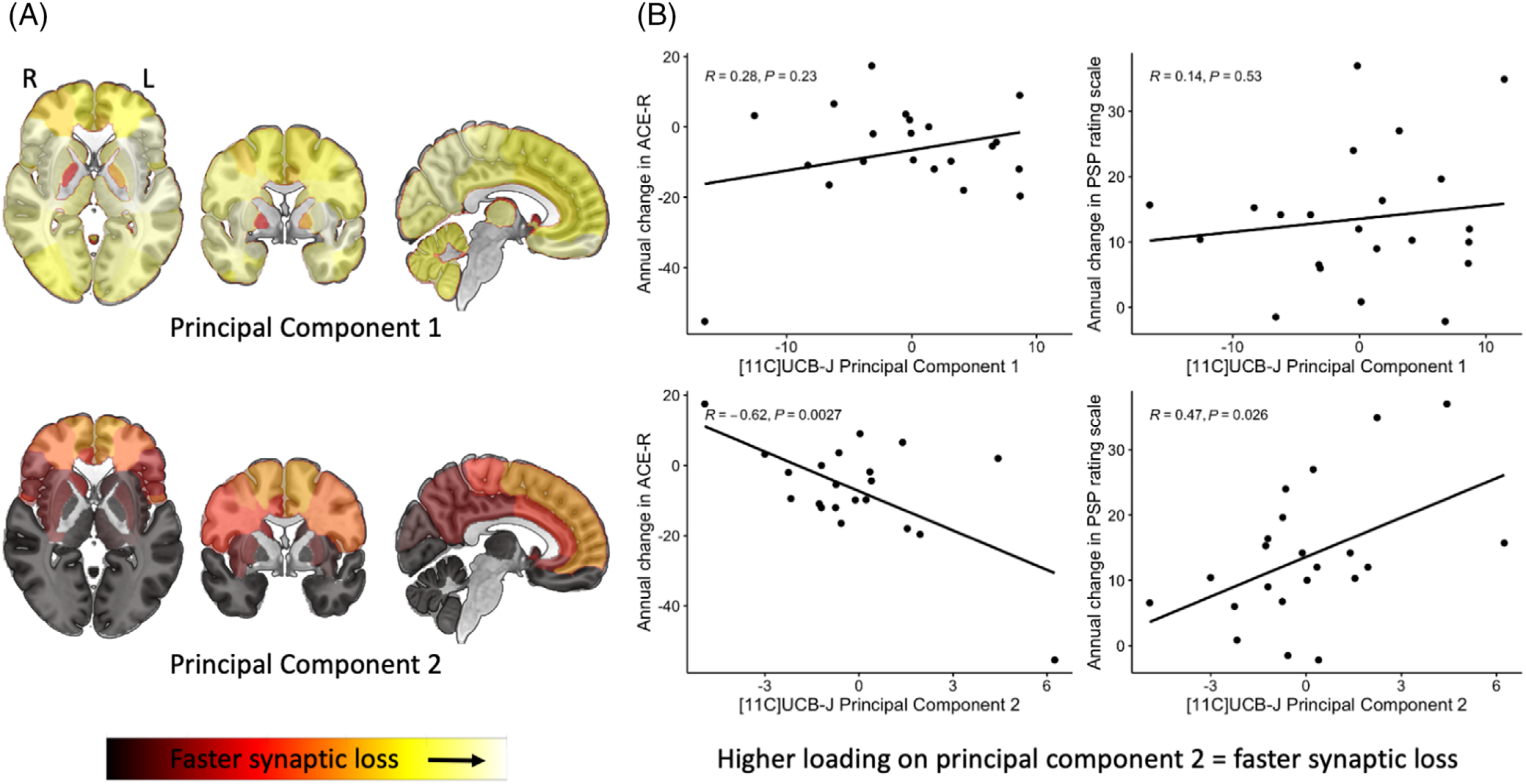
(**A**) Brain maps illustrating principal components 1 and 2 weighting (yellow = faster synaptic loss) obtained from a principal‐component analysis of the annualized proportional change in [^11^C]UCB‐J BP_ND_. (**B**) Scatterplots showing the relationship between symptom severity (progressive supranuclear palsy [PSP] Rating Scale)/Addenbrooke's Cognitive Examination–Revised (ACE‐R) and individual participant loadings on principal components 1 (top row) and 2 (bottom row). [Color figure can be viewed at wileyonlinelibrary.com]

We identified two principal components (with an eigenvalue >1), accounting for 80% of the variance in the annual change in regional [^11^C]UCB‐J BP_ND_. The first component constitutes a global reduction in [^11^C]UCB‐J BP_ND_ across the brain. The second component loads onto the frontal lobe, cingulate, and the postcentral gyrus. Principal‐component weightings projected on brain maps are shown in Figure 4 (and in Supporting Information Fig. S2 in Data S1 for BP_ND_ without PVC).

We applied a general linear model to individual loadings on principal components and the annual change in the PSP Rating Scale/ACE‐R scores. We found that individuals with higher loading on principal component two (therefore faster synaptic loss within the frontal lobe, motor strip, and cingulate regions) had a faster progression on the PSP Rating Scale (*R* = 0.47, *P* = 0.03) and faster decline in ACE‐R scores (*R* = −0.62, *P* = 0.003; scatterplots in Fig. 4; Supporting Information Fig. S2 in Data S1). We also applied a robust general linear model to downweigh the effect of outlying individuals and obtained the same significant correlation. Repeating this approach with gray matter volume, we identified five principal components collectively explaining more than 80% variance in the volumetric data, but none correlated with a progression in the PSP Rating Scale or the ACE‐R, echoing our voxelwise findings.

## 4 Discussion

There are three main findings of this study. First, there is widespread severe synaptic loss in patients with PSP and CBD (inferred in vivo from amyloid‐negative CBS). Second, synaptic loss progresses markedly even over 1 year, with 3% to 4% reduction in the frontal lobe and caudate nucleus. Third, this longitudinal synaptic loss correlates with clinical decline in cognition and global clinical severity.

The widespread synaptic loss in the current 48 patients affirms the results of a previous smaller cross‐sectional cohort.^17^ Synaptic loss is seen both in regions affected in early stages of disease (eg, basal ganglia, thalamus, and frontal lobe) and in those affected in later stages (eg, occipital lobe), and it is more severe and extensive than MRI measures of gray matter atrophy. This study goes beyond the former work in the longitudinal phase. We observed an average increase of 13.6 points in the PSP Rating Scale, similar to previous longitudinal studies of PSP.^2^, ^34^ Based on an average baseline symptom duration of 4 to 6 years and a mean baseline synaptic reduction of ~11% to 25% (vs. controls), we predicted a 3% to 4% annual synaptic loss. This was confirmed in the longitudinal cohort, at least for the caudate and frontal cortex. In the Kovacs staging of tau pathology in PSP, neuronal pathology progresses from pallidum, midbrain tegmentum and pons, then striatum, and only in stage 4 in the prefrontal cortex. Astroglial pathology also affects the frontal cortex and thalamus by stage 3.^9^ Synaptic loss follows a similar pattern of reduction, with the most severe reductions seen in the basal ganglia, thalamus, and frontal lobes; our longitudinal observations are consistent with this spatiotemporal progression of pathology.

Synaptic loss is a common convergence point in many neurodegenerative proteinopathies with in vivo evidence available in people with frontotemporal dementia,^35^, ^36^ Alzheimer's disease,^37^, ^38^ Lewy body dementia, and Parkinson's disease,^39^ correlating with worse performance on cognitive tasks. The evidence for progressive in vivo synaptic loss, as measured with longitudinal [^11^C]UCB‐J PET imaging, is however limited. Vanderlinden and colleagues^40^ have recently reported significant progressive reductions (6%–9%) in patients with amnestic mild cognitive impairment, over 2 years, correlating with cognitive decline. Venkataraman and colleagues^41^ observed reduced baseline [^11^C]UCB‐J binding potential in disease‐specific areas in eight patients with Alzheimer's disease, but they did not find significant progressive loss over 12 to 18 months of follow‐up. Similarly, although Delva and colleagues^42^ illustrate significant baseline synaptic loss within the substantia nigra of 27 patients with early and mild Parkinson's disease, they did not observe significant progression over 2 years despite deteriorations in motor symptoms.

There are several explanations for the variability in the findings between our study in PSP/CBD and studies of other dementias. First, synaptic loss is related to severity of clinical signs and symptoms; people with PSP and CBD have marked cognitive deficits and rapid progression.^1^, ^2^ For other disorders with either normal cortical synaptic density at baseline and/or slow clinical progression, the power to detect longitudinal change will be greatly reduced. For example, the cohort of people with (mild and early) Parkinson's disease in Delva and colleagues' study^42^ did not have significant cortical synaptic loss or cognitive symptoms at baseline or at follow‐up, unlike people with Lewy body dementia and Parkinson's disease with dementia who show significant baseline cortical synaptic loss.^43^ In Alzheimer's disease, cognitive symptoms are prominent^44^ and correlate with baseline synaptic loss.^37^ However, in Venkataraman and colleagues'^41^ cohort of eight people with Alzheimer's disease, although [^11^C]UCB‐J binding was reduced in all examined regions of interest at follow‐up, none was statistically significant, likely because of a limited sample size.

Second, there are technical issues in measuring change in [^11^C]UCB‐J binding over time. Our study uses the simplified tissue reference model to determine BP_ND_ of [^11^C]UCB‐J. Although in a recent test–retest validation study this method is reported as the most optimal to obtain quantitatively accurate and repeatable parametric images for [^11^C]UCB‐J binding, Tuncel and colleagues^45^, ^46^ report high regional variability in test–retest measures in healthy volunteers and patients alike. This variance may therefore explain differential sensitivity to progression in diseases that vary in their principal functional anatomical distributions. Even when expected mean changes in synaptic density are of the order of 3% to 4%, low reliability will greatly reduce power to detect within‐subject decline. The midbrain, a key area affected by PSP pathology,^9^ is particularly affected by this technical limitation. The applicability of [^11^C]UCB‐J imaging to the midbrain therefore raises interesting issues. The gray matter volumes in the midbrain are small compared with other gray matter dense brain regions that are affected by PSP/CBD (eg, basal ganglia, thalamus, cortex). One result is that gray matter segmentation of this region is challenging, which in turn affects the midbrain‐derived [^11^C]UCB‐J binding potential. Given this, we expect variability in our binding potentials between patients and within patients across longitudinal assessment. At a group level, the effect size for synaptic reduction compared with controls is large despite the challenges posed by the variability cross‐sectionally. Progressive midbrain atrophy, a hallmark imaging finding in PSP,^34^ includes both reduced gray matter volume and significant loss of white matter integrity and volume. The observed lack of change in midbrain synaptic density over time (Supporting Information Tables S1 and S3 in Data S1) may well be related to low signal‐to‐noise ratio rather than true stability in synaptic density (high variability in the synaptic signal from gray matter and small expected percentage change in synaptic density over time).

Although we observed significant gray matter atrophy over time, our principal‐component analysis and voxelwise analysis show that faster loss of synapses within the frontal lobe, but not gray matter volume, correlates with progression of symptoms. That synaptic loss is better correlated with symptom progression and severity than gray matter volume echoes postmortem findings in the 4R primary tauopathies and human Alzheimer's disease tauopathy,^47^, ^48^, ^49^ as well as in animal models of tauoapathy.^50^

There are limitations to our study. First, our longitudinal cohort is modest in size (n = 22), and delays resulting from the COVID‐19 pandemic increased the scanning interval from 12 to 16 months and increased dropout of participants. Despite this, our 22 patients are similar in their baseline characteristics to those in the baseline‐only study. This reduces the potential bias toward patients with milder disease. Second, clinical diagnostic criteria for PSP–Richardson's syndrome and amyloid‐negative CBS (here inferred as CBD) were used to select a clinical cohort with likely a 4R‐tauopathy as the underlying pathological diagnosis. Although both PSP–Richardson's syndrome and amyloid‐negative CBS are highly correlated with 4R‐tauopathy at postmortem,^7^, ^51^, ^52^ other pathologies are possible. Third, in PET studies of neurodegeneration with atrophy, gray matter volume loss can affect the interpretation of PET signals. However, synaptic loss in PSP and CBD occurs even in areas of the brain without discernible atrophy on MRI.^17^, ^53^ Nonetheless, we used PVC to minimize the effect of atrophy on binding estimates and obtained similar results in all the main analyses (Supporting Information Fig. S1, Fig. 3, and Table S3 in Data S1).

In conclusion, we found severe and progressive synaptic loss in a cohort of patients with PSP and amyloid‐negative CBS. We confirmed our prediction that synaptic loss occurs at a rate of ~3% to 4% per year in disease‐related areas. The individual variability in the rate of synaptic loss is associated with how quickly patients deteriorate over time. Future studies are required to examine the in vivo relationship between longitudinal changes in synaptic loss and the temporal distribution of tau pathology. With the pathogenic conversion of toxic oligomers, mitochondrial stress, and inflammation on synaptic toxicity, we propose that synaptic loss is a functionally relevant intermediate marker of disease severity and suitable as either a target or an outcome measure in future trials of disease‐modifying agents in PSP/CBD.

## Financial Disclosures

N.H. is funded by National Institute for Health Research and the Association of British Neurologists—Patrick Berthoud Charitable Trust. P.S.J., G.S., and M.N. have no financial disclosures to report. M.M. is funded by the Alzheimer's Research UK Race Against Dementia Fellowship and has no financial disclosures to report. D.J.W., D.S., P.S., Y.T.H., T.D.F., E.M., and K.P.B. have no financial disclosures to report. T.R. received funding from the Cambridge Center for Parkinson‐Plus, the Cambridge NIHR Biomedical Research Center, UK Alzheimer's Society, and the Medical Research Center Sprint Fund. J.T.O. provides consultancy to Alliance Medical, Merck, Roche, and Biogen; he has received honoraria from GE Healthcare and is the Research Strategy Council Chair at the UK Alzheimer's Society. J.B.R. serves as an associate editor to *Brain* and is a non‐remunerated trustee of the Guarantors of Brain and the PSP Association (UK). He provides consultancy to Asceneuron, Astex, Curasen, WAVE, Cumulus, and UCB.

## Author Roles

N.H., J.T.O., and J.B.R. contributed to the conception and design of the study. N.H. was involved in data acquisition, analysis, and drafting the manuscript, and prepared the figures and tables. P.S.J., G.S., M.N., M.M., D.J.W., D.S., T.D.F., Y.T.H., and T.R. contributed to data acquisition and edited the final version of the manuscript draft. F.I.A., E.M., K.B., J.T.O., and J.B.R. edited the final version of the manuscript.

## Supporting information


**Data S1.** Supporting Information.

## Data Availability

The data that support the findings of this study are available from the corresponding author, upon reasonable request. Raw data and clinical data requests may be subject to restrictions required to preserve participant confidentiality. A data transfer agreement may be required.
