# Supplementary material for: Longitudinal Synaptic Loss in Primary Tauopathies: An In Vivo [ 11C]UCB‐J Positron Emission Tomography Study
Source: Mov Disord. 2023 May 12;38(7):1316–26. doi: 10.1002/mds.29421 (PMC10947001; doi:10.1002/mds.29421)
Supplement: Supplementary file 1 — Data S1. Supporting Information. [file MDS-38-1316-s001.docx]

**Longitudinal synaptic loss in primary tauopathies:**

**an *in vivo* [^11^C]UCB-J PET study**

***Supplementary material***

## Longitudinal change in [^11^C]UCB-J BP_ND_ (CSF corrected) over one year (%) in Hammersmith atlas regions of interest

| **Region** | **Mean** | **SD** | **Min** | **Max** | **p value** |
| --- | --- | --- | --- | --- | --- |
| Pallidum L | -4.2 | 10.6 | -20.8 | 25.2 | 0.080 |
| Caudate nucleus R | -3.9 | 9.9 | -23.7 | 13.1 | **0.046** |
| Presubgenual frontal cortex L | -3.5 | 7.9 | -17.9 | 11.6 | **0.030** |
| Presubgenual frontal cortex R | -3.2 | 11.3 | -23.2 | 31.3 | ns |
| Middle frontal gyrus R | -2.7 | 7.3 | -14.1 | 14.2 | ns |
| Caudate nucleus L | -2.6 | 10.4 | -20.1 | 22.4 | ns |
| Superior frontal gyrus R | -2.5 | 8.5 | -19.5 | 14.4 | ns |
| Middle frontal gyrus L | -2.5 | 9.4 | -19.8 | 17.5 | ns |
| Subcallosal area L | -2.3 | 13.2 | -21.0 | 25.2 | ns |
| Hippocampus R | -2.1 | 11.5 | -18.0 | 26.4 | ns |
| Thalamus R | -2.0 | 7.2 | -17.0 | 14.4 | ns |
| Superior frontal gyrus L | -1.9 | 7.8 | -15.6 | 15.8 | ns |
| Inferiolateral remainder of parietal lobe L | -1.8 | 7.9 | -22.5 | 11.7 | ns |
| Cuneus R | -1.8 | 7.3 | -14.2 | 15.7 | ns |
| Subgenual frontal cortex L | -1.7 | 8.4 | -15.2 | 20.2 | ns |
| Thalamus L | -1.7 | 7.2 | -15.7 | 15.3 | ns |
| Inferior frontal gyrus R | -1.7 | 6.6 | -11.8 | 10.6 | ns |
| Anterior orbital gyrus R | -1.7 | 8.5 | -17.5 | 17.8 | ns |
| Inferior frontal gyrus L | -1.7 | 8.3 | -15.3 | 14.1 | ns |
| Superior parietal gyrus R | -1.6 | 7.7 | -22.9 | 11.0 | ns |
| Cuneus L | -1.6 | 8.5 | -15.0 | 24.1 | ns |
| Cingulate gyrus anterior part L | -1.5 | 7.9 | -14.0 | 14.8 | ns |
| Precentral gyrus L | -1.4 | 8.1 | -18.1 | 13.2 | ns |
| Putamen R | -1.4 | 7.5 | -17.2 | 13.5 | ns |
| Subcallosal area R | -1.4 | 14.0 | -18.0 | 46.1 | ns |
| Hippocampus L | -1.3 | 8.2 | -13.6 | 15.6 | ns |
| Fusiform gyrus R | -1.2 | 9.3 | -15.4 | 21.3 | ns |
| Inferiolateral remainder of parietal lobe R | -1.1 | 6.9 | -11.5 | 12.1 | ns |
| Subgenual frontal cortex R | -1.1 | 13.7 | -27.2 | 28.2 | ns |
| Insula R | -1.1 | 8.3 | -18.3 | 11.9 | ns |
| Amygdala R | -1.0 | 9.4 | -16.0 | 22.5 | ns |
| Superior parietal gyrus L | -0.9 | 8.3 | -20.8 | 14.4 | ns |
| Parahippocampal and ambient gyri R | -0.8 | 6.6 | -18.2 | 11.4 | ns |
| Superior temporal gyrus posterior part R | -0.8 | 8.8 | -13.0 | 17.7 | ns |
| Cingulate gyrus anterior part R | -0.8 | 9.3 | -21.2 | 22.4 | ns |
| Cerebellum gm L | -0.8 | 8.5 | -12.8 | 25.9 | ns |
| Precentral gyrus R | -0.7 | 8.0 | -13.3 | 14.9 | ns |
| Lateral remainder of occipital lobe L | -0.7 | 8.1 | -13.7 | 18.9 | ns |

| **Region** | **Mean** | **SD** | **Min** | **Max** | **p value** |
| --- | --- | --- | --- | --- | --- |
| Amygdala L | -0.6 | 10.5 | -18.8 | 15.9 | ns |
| Anterior orbital gyrus L | -0.6 | 10.1 | -18.4 | 28.9 | ns |
| Putamen L | -0.6 | 8.0 | -14.6 | 16.6 | ns |
| Lateral remainder of occipital lobe R | -0.6 | 6.7 | -10.7 | 12.4 | ns |
| Cerebellum gm R | -0.6 | 7.5 | -12.4 | 18.3 | ns |
| Superior temporal gyrus posterior part L | -0.5 | 8.9 | -17.4 | 18.5 | ns |
| Straight gyrus L | -0.5 | 10.7 | -21.8 | 30.3 | ns |
| Middle and inferior temporal gyrus R | -0.4 | 8.9 | -14.6 | 26.3 | ns |
| Lingual gyrus L | -0.3 | 7.6 | -11.0 | 14.2 | ns |
| Posterior orbital gyrus R | -0.2 | 9.1 | -14.5 | 19.7 | ns |
| Anterior temporal lobe medial part L | -0.1 | 9.9 | -14.6 | 25.9 | ns |
| Postcentral gyrus L | -0.1 | 8.0 | -17.2 | 17.4 | ns |
| Superior temporal gyrus anterior part L | 0.0 | 9.8 | -14.1 | 20.3 | ns |
| Lingual gyrus R | 0.0 | 7.5 | -11.2 | 15.9 | ns |
| Pallidum R | 0.1 | 8.4 | -14.6 | 14.9 | ns |
| Posterior temporal lobe L | 0.2 | 10.3 | -16.1 | 33.6 | ns |
| Anterior temporal lobe medial part R | 0.3 | 10.0 | -14.0 | 29.1 | ns |
| Fusiform gyrus L | 0.3 | 11.1 | -18.9 | 34.4 | ns |
| Parahippocampal and ambient gyri L | 0.4 | 12.3 | -19.3 | 26.4 | ns |
| Posterior temporal lobe R | 0.4 | 8.4 | -11.2 | 19.7 | ns |
| Superior temporal gyrus anterior part R | 0.4 | 11.7 | -16.9 | 29.4 | ns |
| Gyrus cinguli posterior part L | 0.5 | 6.5 | -12.3 | 15.6 | ns |
| Gyrus cinguli posterior part R | 0.5 | 8.1 | -21.2 | 12.0 | ns |
| Posterior orbital gyrus L | 0.5 | 10.5 | -17.3 | 27.9 | ns |
| Cerebellum dentate L | 0.5 | 11.8 | -25.3 | 27.4 | ns |
| Insula L | 0.6 | 9.5 | -15.5 | 24.6 | ns |
| Straight gyrus R | 0.7 | 10.5 | -14.6 | 35.2 | ns |
| Middle and inferior temporal gyrus L | 0.7 | 11.9 | -13.9 | 45.1 | ns |
| Medial orbital gyrus R | 0.7 | 9.6 | -17.0 | 25.4 | ns |
| Anterior temporal lobe lateral part R | 0.9 | 13.9 | -23.9 | 50.9 | ns |
| Postcentral gyrus R | 0.9 | 8.6 | -12.0 | 17.2 | ns |
| Anterior temporal lobe lateral part L | 0.9 | 13.8 | -23.7 | 46.8 | ns |
| Lateral orbital gyrus R | 1.1 | 9.4 | -12.9 | 26.3 | ns |
| Lateral orbital gyrus L | 1.6 | 10.7 | -17.3 | 26.2 | ns |
| Medial orbital gyrus L | 1.8 | 9.9 | -13.0 | 32.5 | ns |
| Cerebellum dentate R | 2.8 | 14.1 | -23.1 | 44.7 | ns |

Supplementary Table 1. Annual percentage change (Mean, standard deviation (SD), minimum (Min) and maximum (Max)), in [^11^C]UCB-J BP_ND_ (CSF corrected). Unadjusted p values obtained from a two-way ANOVA of regional [^11^C]UCB-J BP_ND_ at baseline and follow-up. L: left, R: right.

## Longitudinal change in grey matter volume over one year (%) in Hammersmith atlas regions of interest

| **Region** | **Mean** | **SD** | **Min** | **Max** | **p value** | **adjusted p value** |
| --- | --- | --- | --- | --- | --- | --- |
| Thalamus_R | -11.8 | 10.6 | -36.9 | 1.8 | **0.000** | **0.002** |
| Precentral_gyrus_L | -7.1 | 7.2 | -25.4 | 4.8 | **0.000** | **0.004** |
| Caudate_nucleus_R | -7.5 | 7.5 | -24.4 | 5.7 | **0.000** | **0.009** |
| Inferiolateral_remainder_of_parietal_lobe_R | -5.2 | 5.1 | -15.6 | 3.1 | **0.001** | **0.043** |
| Cerebellum_gm_L | -2.8 | 4.0 | -13.5 | 5.6 | **0.001** | **0.044** |
| Thalamus_L | -8.8 | 12.4 | -36.2 | 22.8 | **0.001** | ns |
| Inferior_frontal_gyrus_L | -6.3 | 8.9 | -29.5 | 8.9 | **0.001** | ns |
| Cuneus_L | -6.5 | 9.6 | -28.0 | 10.0 | **0.004** | ns |
| Superior_temporal_gyrus_anterior_part_R | -4.4 | 7.5 | -25.8 | 5.8 | **0.005** | ns |
| Superior_temporal_gyrus_posterior_part_R | -3.4 | 5.1 | -14.2 | 4.9 | **0.005** | ns |
| Cuneus_R | -5.8 | 9.1 | -26.4 | 11.9 | **0.006** | ns |
| Precentral_gyrus_R | -5.8 | 10.3 | -35.5 | 10.4 | **0.006** | ns |
| Lingual_gyrus_L | -5.3 | 7.4 | -25.8 | 4.6 | **0.006** | ns |
| Inferior_frontal_gyrus_R | -4.2 | 5.7 | -18.2 | 7.0 | **0.007** | ns |
| Inferiolateral_remainder_of_parietal_lobe_L | -3.8 | 6.1 | -13.7 | 5.9 | **0.009** | ns |
| Posterior_temporal_lobe_R | -2.6 | 4.6 | -12.2 | 7.4 | **0.011** | ns |
| Superior_temporal_gyrus_anterior_part_L | -4.5 | 10.7 | -27.2 | 25.4 | **0.012** | ns |
| Superior_parietal_gyrus_R | -2.6 | 5.0 | -12.2 | 9.6 | **0.012** | ns |
| Lingual_gyrus_R | -2.3 | 4.5 | -14.9 | 5.9 | **0.012** | ns |
| Postcentral_gyrus_R | -3.4 | 8.8 | -22.0 | 21.2 | **0.013** | ns |
| Anterior_temporal_lobe_lateral_part_R | -5.6 | 13.2 | -34.0 | 27.9 | **0.037** | ns |
| Anterior_temporal_lobe_medial_part_L | -5.0 | 10.9 | -29.7 | 14.0 | **0.016** | ns |
| Anterior_temporal_lobe_lateral_part_L | -4.1 | 13.0 | -34.0 | 19.3 | ns | ns |
| Middle_frontal_gyrus_L | -4.1 | 9.8 | -27.5 | 18.8 | **0.017** | ns |
| Middle_frontal_gyrus_R | -4.0 | 9.8 | -27.9 | 11.3 | ns | ns |
| Lateral_orbital_gyrus_L | -4.0 | 12.2 | -19.9 | 30.0 | **0.045** | ns |
| Caudate_nucleus_L | -3.8 | 9.4 | -19.4 | 27.8 | **0.020** | ns |
| Anterior_orbital_gyrus_L | -3.8 | 15.3 | -32.2 | 47.0 | **0.046** | ns |
| Superior_temporal_gyrus_posterior_part_L | -3.4 | 6.9 | -15.8 | 9.9 | **0.029** | ns |
| Posterior_orbital_gyrus_L | -3.3 | 7.9 | -18.2 | 12.5 | **0.041** | ns |
| Putamen_L | -3.3 | 9.4 | -17.0 | 22.9 | **0.021** | ns |
| Subcallosal_area_L | -3.3 | 14.5 | -47.3 | 22.1 | ns | ns |
| Superior_frontal_gyrus_L | -3.3 | 7.1 | -19.8 | 12.2 | **0.016** | ns |
| Middle_and_inferior_temporal_gyrus_R | -3.2 | 6.5 | -21.3 | 5.9 | **0.015** | ns |
| Fusiform_gyrus_L | -3.1 | 6.1 | -16.9 | 4.6 | **0.033** | ns |
| Lateral_remainder_of_occipital_lobe_R | -3.1 | 8.6 | -25.3 | 8.9 | ns | ns |
| Parahippocampal_and_ambient_gyri_L | -3.1 | 7.2 | -25.3 | 8.4 | **0.025** | ns |
| Posterior_orbital_gyrus_R | -2.9 | 5.8 | -18.5 | 5.3 | **0.022** | ns |

| **Region** | **Mean** | **SD** | **Min** | **Max** | **p value** | **adjusted p value** |
| --- | --- | --- | --- | --- | --- | --- |
| Gyrus_cinguli_posterior_part_L | -2.9 | 5.7 | -18.8 | 2.4 | **0.016** | ns |
| Putamen_R | -2.9 | 11.2 | -21.1 | 24.7 | ns | ns |
| Postcentral_gyrus_L | -2.9 | 8.5 | -19.6 | 16.7 | **0.022** | ns |
| Middle_and_inferior_temporal_gyrus_L | -2.8 | 9.1 | -24.4 | 10.0 | ns | ns |
| Gyrus_cinguli_posterior_part_L | -2.9 | 5.7 | -18.8 | 2.4 | **0.016** | ns |
| Amygdala_L | -2.7 | 7.7 | -32.2 | 8.5 | ns | ns |
| Superior_frontal_gyrus_R | -2.6 | 8.3 | -18.9 | 17.4 | ns | ns |
| Anterior_temporal_lobe_medial_part_R | -2.3 | 8.5 | -16.8 | 21.3 | ns | ns |
| Lateral_remainder_of_occipital_lobe_L | -2.1 | 4.7 | -8.7 | 8.0 | ns | ns |
| Anterior_orbital_gyrus_R | -2.0 | 12.1 | -18.0 | 32.9 | ns | ns |
| Posterior_temporal_lobe_L | -1.8 | 3.6 | -10.2 | 3.2 | **0.027** | ns |
| Insula_R | -1.7 | 5.4 | -14.2 | 9.5 | ns | ns |
| Amygdala_R | -1.6 | 5.0 | -12.6 | 8.4 | ns | ns |
| Straight_gyrus_R | -1.4 | 6.9 | -10.0 | 21.7 | ns | ns |
| Superior_parietal_gyrus_L | -1.4 | 5.6 | -13.5 | 15.4 | ns | ns |
| Cerebellum_gm_R | -1.4 | 5.3 | -8.2 | 17.9 | **0.041** | ns |
| Hippocampus_L | -1.3 | 6.0 | -13.7 | 11.7 | ns | ns |
| Straight_gyrus_L | -1.2 | 6.8 | -9.1 | 16.5 | ns | ns |
| Fusiform_gyrus_R | -0.9 | 6.3 | -17.9 | 11.4 | ns | ns |
| Gyrus_cinguli_posterior_part_R | -0.7 | 5.2 | -13.5 | 9.4 | ns | ns |
| Insula_L | -0.6 | 5.8 | -14.4 | 16.8 | ns | ns |
| Parahippocampal_and_ambient_gyri_R | -0.1 | 7.1 | -12.5 | 18.2 | ns | ns |
| Lateral_orbital_gyrus_R | 0.0 | 9.1 | -16.8 | 19.5 | ns | ns |
| Medial_orbital_gyrus_R | 0.1 | 8.6 | -10.8 | 27.6 | ns | ns |
| Hippocampus_R | 0.2 | 3.9 | -6.5 | 9.3 | ns | ns |
| Subgenual_frontal_cortex_L | 0.5 | 9.6 | -17.0 | 32.4 | ns | ns |
| Subgenual_frontal_cortex_R | 0.8 | 15.0 | -36.1 | 33.6 | ns | ns |
| Cingulate_gyrus_anterior_part_L | 1.9 | 17.3 | -30.2 | 66.8 | ns | ns |
| Subcallosal_area_R | 2.6 | 24.2 | -72.2 | 67.7 | ns | ns |
| Medial_orbital_gyrus_L | 3.0 | 12.8 | -15.1 | 35.2 | ns | ns |
| Presubgenual_frontal_cortex_L | 3.9 | 21.2 | -52.1 | 59.0 | ns | ns |
| Cingulate_gyrus_anterior_part_R | 7.9 | 22.0 | -12.5 | 88.2 | ns | ns |
| Presubgenual_frontal_cortex_R | 8.0 | 34.4 | -73.7 | 120.5 | ns | ns |

Supplementary Table 2. Annual percentage change in grey matter volume (Mean, standard deviation (SD), minimum (Min) and maximum (Max). The significant grey matter volumetric changes are highlighted in bold. L: left, R: right.

**Widespread severe reductions in [^11^C]UCB-J BP_ND_ without partial volume correction, beyond grey matter atrophy in the cross-sectional cohort**


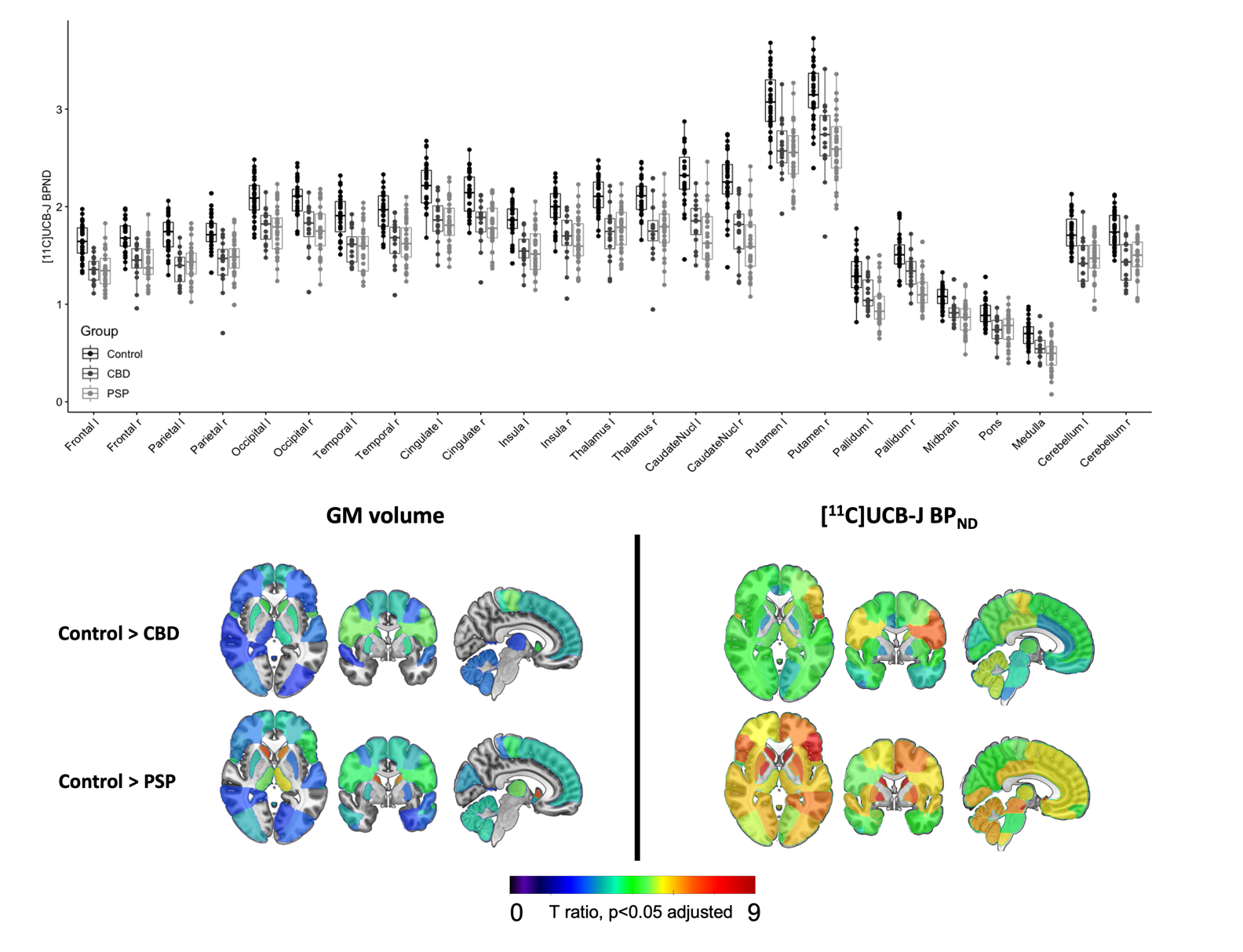


**A**

**B**

*Supplementary Figure 1.* A) Regional [^11^C]UCB-J BP_ND_ without partial volume correction in healthy volunteers (n=31) and patients (PSP, n=32; CBD, n=16), in major regions of interest. B) t-statistic maps comparing grey matter (GM) volume and [^11^C]UCB-J BP_ND_ in patients versus controls. Only t values significant at p<0.05 adjusted for multiple comparisons, are shown here; higher t values illustrate greater reduction in GM volume and synaptic density; orange/red=more severe atrophy/synaptic loss).

## Longitudinal change in [^11^C]UCB-J BP_ND_ (CSF uncorrected) over one year (%)

A one-sample t-test, applied to the mean proportional change in [^11^C]UCB-J BP_ND_ without partial volume correction over one year, suggested a significant overall reduction in [^11^C]UCB-J BP_ND_ over time (p=0.002). A paired-sample, two-way ANOVA, testing for a difference in [^11^C]UCB-J BP_ND_ between baseline and follow-up visits, showed a significant region-by-visit interaction (p=0.002). A post-hoc, paired-sample ANOVA in each region between visits, illustrated significant reductions in [^11^C]UCB-J BP_ND_ within the right caudate (-5.6%, p=0.01), left presubgenual frontal cortex (-3.7%, p=0.05), unadjusted. A summary of annual percentage change in [^11^C]UCB-J BP_ND_ in all relevant Hammersmith atlas regions of interest are given in Supplementary Table 3.

| **Region** | **Mean** | **SD** | **Min** | **Max** |
| --- | --- | --- | --- | --- |
| Caudate_nucleus_R | **-5.6** | **9.8** | **-22.5** | **13.5** |
| Hippocampus_R | -3.9 | 11.2 | -20.9 | 22.7 |
| Hippocampus_L | -3.9 | 8.6 | -16.8 | 12.8 |
| Pallidum_L | -3.9 | 10.8 | -21.4 | 25.2 |
| Subcallosal_area_R | -3.8 | 10.1 | -20.4 | 10.9 |
| Presubgenual_frontal_cortex_L | -3.7 | 8.9 | -17.6 | 13.9 |
| Caudate_nucleus_L | -3.4 | 11.4 | -23.8 | 24.1 |
| Subcallosal_area_L | -3.1 | 15.2 | -23.8 | 35.5 |
| Fusiform_gyrus_R | -3.0 | 9.5 | -20.9 | 12.9 |
| Middle_frontal_gyrus_L | -2.7 | 9.9 | -19.7 | 20.6 |
| Cuneus_R | -2.6 | 7.8 | -15.0 | 15.1 |
| Fusiform_gyrus_L | -2.6 | 9.4 | -19.4 | 17.8 |
| Thalamus_R | -2.4 | 7.5 | -18.3 | 14.0 |
| Anterior_temporal_lobe_lateral_part_L | -2.2 | 9.3 | -18.9 | 15.7 |
| Superior_frontal_gyrus_R | -2.1 | 9.3 | -19.7 | 18.8 |
| Middle_frontal_gyrus_R | -2.1 | 8.3 | -16.2 | 18.5 |
| Lateral_remainder_of_occipital_lobe_L | -2.1 | 8.2 | -15.2 | 17.5 |
| Presubgenual_frontal_cortex_R | -2.1 | 12.6 | -20.2 | 44.7 |
| Thalamus_L | -2.1 | 7.3 | -16.8 | 15.6 |
| Inferiolateral_remainder_of_parietal_lobe_L | -2.1 | 9.2 | -25.6 | 15.9 |
| Parahippocampal_and_ambient_gyri_R | -2.1 | 7.1 | -21.8 | 8.7 |
| Cuneus_L | -2.1 | 7.9 | -14.4 | 15.2 |
| Cerebellum_gm_L | -2.1 | 7.9 | -15.9 | 20.7 |
| Anterior_temporal_lobe_medial_part_L | -2.0 | 8.8 | -17.0 | 13.9 |
| Middle_and_inferior_temporal_gyrus_L | -1.9 | 9.0 | -16.8 | 25.0 |
| Inferior_frontal_gyrus_L | -1.9 | 8.1 | -14.2 | 16.2 |
| Anterior_orbital_gyrus_L | -1.9 | 8.5 | -20.4 | 15.6 |
| Anterior_orbital_gyrus_R | -1.9 | 8.0 | -15.7 | 16.3 |
| Insula_R | -1.9 | 8.6 | -20.1 | 13.8 |
| Amygdala_R | -1.8 | 9.8 | -19.0 | 22.3 |
| Superior_frontal_gyrus_L | -1.7 | 8.4 | -14.8 | 16.6 |
| Posterior_temporal_lobe_L | -1.7 | 10.1 | -21.5 | 27.2 |
| Lingual_gyrus_L | -1.7 | 7.4 | -13.4 | 11.7 |
| Parahippocampal_and_ambient_gyri_L | -1.6 | 12.5 | -23.3 | 19.0 |
| Subgenual_frontal_cortex_L | -1.6 | 8.8 | -14.5 | 21.8 |
| Superior_parietal_gyrus_R | -1.6 | 9.6 | -28.3 | 15.4 |
| Inferior_frontal_gyrus_R | -1.5 | 7.3 | -15.1 | 14.6 |
| Superior_temporal_gyrus_posterior_part_L | -1.5 | 9.3 | -19.4 | 18.8 |
| Posterior_orbital_gyrus_R | -1.5 | 8.3 | -15.4 | 16.9 |
| Superior_temporal_gyrus_anterior_part_L | -1.5 | 8.4 | -17.8 | 18.8 |

| **Region** | **Mean** | **SD** | **Min** | **Max** |
| --- | --- | --- | --- | --- |
| Amygdala_L | -1.5 | 10.2 | -19.7 | 12.8 |
| Cerebellum_gm_R | -1.4 | 7.4 | -15.0 | 14.7 |
| Middle_and_inferior_temporal_gyrus_R | -1.4 | 8.1 | -17.1 | 14.5 |
| Precentral_gyrus_L | -1.3 | 8.8 | -19.8 | 16.8 |
| Inferiolateral_remainder_of_parietal_lobe_R | -1.3 | 7.7 | -14.1 | 11.1 |
| Lingual_gyrus_R | -1.2 | 7.6 | -13.9 | 12.3 |
| Lateral_remainder_of_occipital_lobe_R | -1.2 | 7.6 | -11.9 | 12.5 |
| Superior_temporal_gyrus_posterior_part_R | -1.2 | 9.2 | -14.4 | 20.4 |
| Anterior_temporal_lobe_lateral_part_R | -1.2 | 9.7 | -25.9 | 22.8 |
| Cingulate_gyrus_anterior_part_L | -1.1 | 9.8 | -17.6 | 15.9 |
| Anterior_temporal_lobe_medial_part_R | -1.1 | 9.0 | -15.3 | 19.7 |
| Putamen_R | -1.1 | 7.0 | -12.5 | 13.7 |
| Subgenual_frontal_cortex_R | -1.0 | 13.8 | -20.9 | 32.3 |
| Straight_gyrus_L | -0.9 | 10.1 | -20.1 | 24.4 |
| Posterior_orbital_gyrus_L | -0.9 | 9.2 | -17.0 | 18.2 |
| Lateral_orbital_gyrus_L | -0.9 | 9.0 | -17.9 | 15.9 |
| Medial_orbital_gyrus_R | -0.9 | 9.6 | -18.7 | 20.3 |
| Superior_parietal_gyrus_L | -0.8 | 9.9 | -25.3 | 18.5 |
| Posterior_temporal_lobe_R | -0.7 | 8.7 | -11.7 | 16.2 |
| Precentral_gyrus_R | -0.7 | 9.0 | -16.4 | 17.9 |
| Superior_temporal_gyrus_anterior_part_R | -0.7 | 11.1 | -25.4 | 22.6 |
| Straight_gyrus_R | -0.6 | 9.9 | -15.2 | 27.8 |
| Insula_L | -0.6 | 9.4 | -16.5 | 20.7 |
| Cingulate_gyrus_anterior_part_R | -0.5 | 10.0 | -19.9 | 20.3 |
| Lateral_orbital_gyrus_R | -0.4 | 8.3 | -15.3 | 16.3 |
| Putamen_L | -0.4 | 7.7 | -10.6 | 16.5 |
| Brainstem_mid_B | -0.4 | 8.7 | -14.3 | 23.3 |
| Medial_orbital_gyrus_L | -0.1 | 8.4 | -16.4 | 18.5 |
| Pallidum_R | 0.0 | 7.8 | -14.5 | 14.2 |
| Cerebellum_dentate_L | 0.1 | 11.7 | -23.8 | 25.8 |
| Postcentral_gyrus_L | 0.2 | 9.2 | -20.2 | 22.4 |
| Brainstem_pon_B | 0.3 | 11.9 | -17.4 | 36.1 |
| Gyrus_cinguli_posterior_part_R | 0.3 | 9.2 | -22.2 | 16.8 |
| Gyrus_cinguli_posterior_part_L | 0.6 | 8.0 | -14.0 | 18.1 |
| Postcentral_gyrus_R | 1.2 | 9.4 | -13.3 | 21.0 |
| Cerebellum_dentate_R | 2.4 | 14.1 | -22.5 | 44.2 |
| Brainstem_med_B | 4.7 | 22.5 | -31.3 | 76.2 |

Supplementary Table 3. Annual percentage change (Mean, standard deviation (SD), minimum (Min) and maximum (Max)), in [^11^C]UCB-J BP_ND_ (without partial volume correction). The annual percentage change in the right caudate is significantly reduced (p<0.05, unadjusted). B: bilateral, L: left, R: right.

## Faster reductions in frontal and cingulate [^11^C]UCB-J BP_ND_ is associated with worsening symptoms over time; principal component analysis (without partial volume correction).


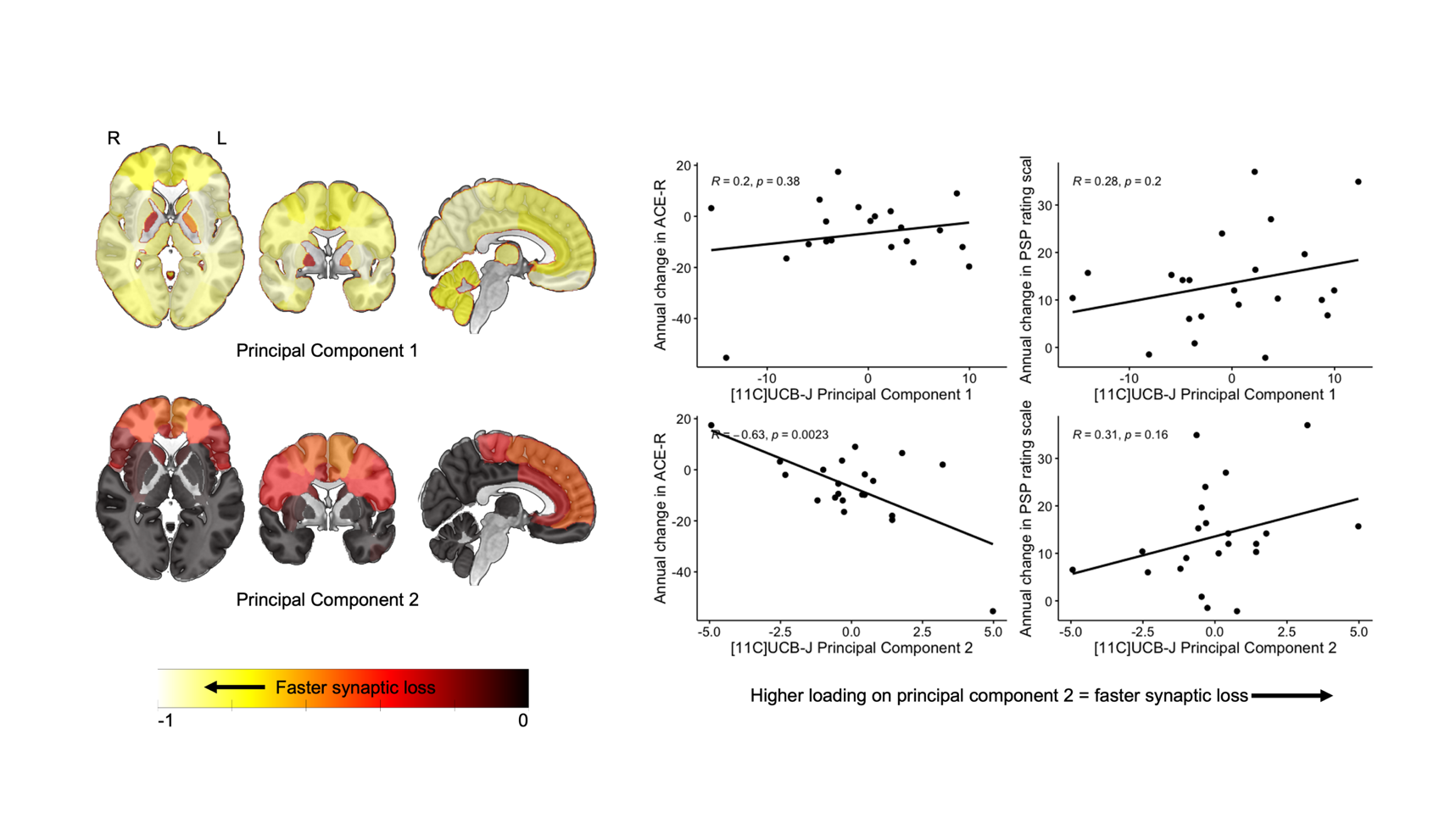

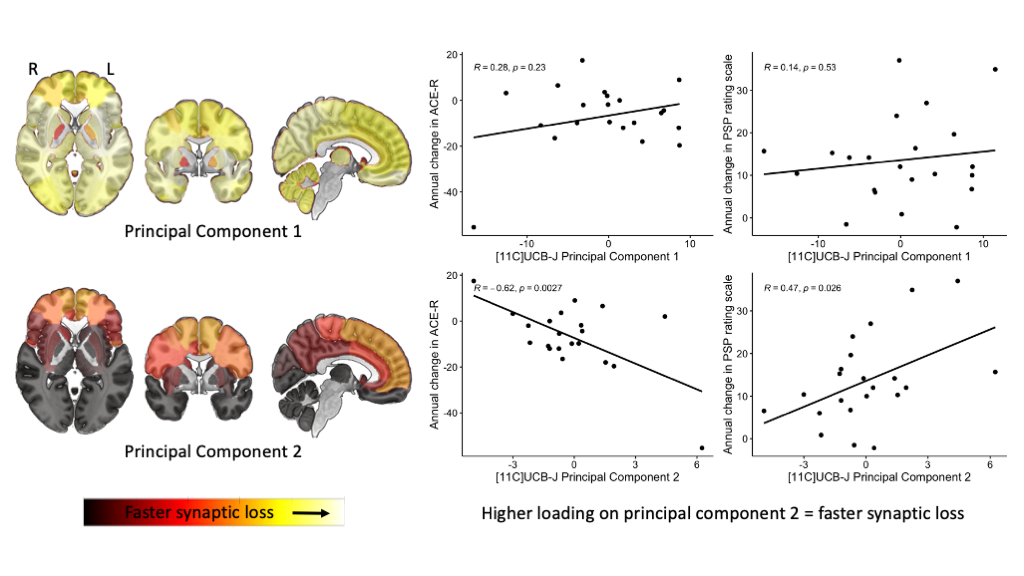

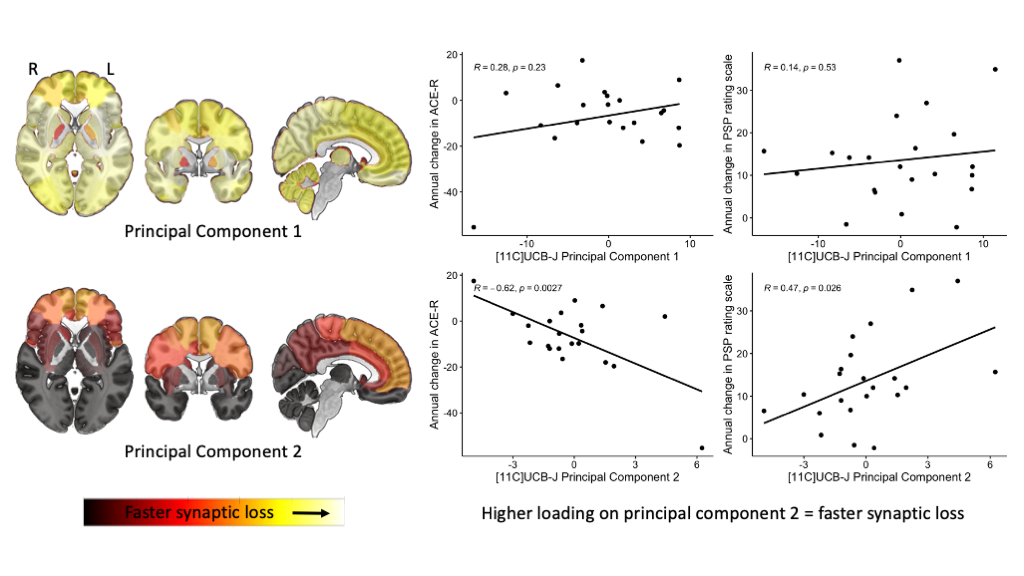


**A**

**B**

*Supplementary Figure 2.* (A) Brain maps illustrating principle component 1 and 2 weighting (yellow = faster synaptic loss) obtained from a principal component analysis of the annualised proportional change in [^11^C]UCB-J BP_ND_ (without partial volume correction). (B) The relationship between disease severity (PSP rating scale)/Addenbrooke’s Cognitive Examination-Revised (ACE-R), and individual loadings on principal components 1 (top row) and 2 (bottom row). L: left, R: right.
